# Atypical *Actinobacillus pleuropneumoniae* serotype 12 strains with a higher virulence potential

**DOI:** 10.1186/s13567-025-01579-9

**Published:** 2025-07-13

**Authors:** Antony T. Vincent, Sonia Lacouture, Guillaume St-Jean, Rodrigo Tapia, Servane Payen, Michiha Kon, Joachim Frey, Ho To, Marcelo Gottschalk

**Affiliations:** 1 Research Group On Infectious Diseases in Production Animals (GREMIP) and Swine and Poultry Infectious Diseases Research Center (CRIPA), Department of Pathology and Microbiology, Faculty of Veterinary Medicine, University of Montreal, Saint-Hyacinthe, QC J2S 2M2 Canada; 2 Virbac Chile, Cerrillos, Santiago, Chile; 3 Université Laval, Québec, QC Canada; 4 University of Bern, Bern, Switzerland; 5 Nippon Institute for Biological Science, Tokyo, Japan; 6 Faculty of Agriculture and Aquaculture, University of Cuu Long, Vinh Long, Vietnam

**Keywords:** *Actinobacillus pleuropneumoniae*, toxins, CPS/LPS combined serotypes, virulence, atypical strains

## Abstract

*Actinobacillus pleuropneumoniae* is the causative agent of porcine pleuropneumonia, a disease of major economic impact. Serotype 12 is generally considered to be of low virulence and is typically associated with subclinical infections. However, we describe four atypical serotype 12 field strains recovered from severe clinical outbreaks in Chile. These strains exhibited an unusual toxin gene profile (*apxIICA*, *apxIIICA*, *apxIBD*, *apxIIIBD*), suggesting the ability to produce both ApxII and ApxIII toxins. Comparative genomic analyses revealed that these atypical strains carry the capsule genes of serotype 12 but the LPS biosynthesis genes of serotype 15, indicating a hybrid genomic structure. Phylogenetic analysis confirmed that they cluster separately from classical serotype 12 strains and are closely related to other atypical strains from Japan, USA, and Canada. Experimental infection in pigs demonstrated significantly increased virulence of the atypical Chilean strain compared to the reference strain of serotype 12, with higher clinical scores, severe lung lesions, and atypical serological responses against serotypes 3/6/8/15/17. These findings challenge the traditional view of serotype 12 as low-virulence and highlight the need for improved diagnostic approaches that incorporate both capsule and LPS profiling. The existence of these atypical strains has important implications for disease surveillance, diagnostics, and vaccine development in swine health management.

**Supplementary Information:**

The online version contains supplementary material available at 10.1186/s13567-025-01579-9.

## Introduction

*Actinobacillus pleuropneumoniae* is the etiologic agent of porcine pleuropneumonia. It is one of the most important swine bacterial respiratory pathogens and is found worldwide [1]. The most virulent strains can rapidly induce fatal fibrinohemorrhagic and necrotizing pleuropneumonia in pigs of all ages. Survivors often have devitalized bacteria-laden sequestra in their lungs. The economic importance of swine pleuropneumonia is mainly due to mortality, reduced growth, veterinary costs (antimicrobials, vaccinations), and condemnations at the slaughterhouse [1].

Various methods have been developed to characterize *A. pleuropneumoniae* strains, primarily to investigate their epidemiological distribution and, to some extent, assess their virulence. Serotyping has traditionally been the preferred approach for this characterization. The identification of serotypes relies on antigenic and structural variations in capsular polysaccharides (CPS) or the genes responsible for their biosynthesis [2]. There are 19 serotypes described so far, although serotypes 9/11 are highly difficult to differentiate. Indeed, a comprehensive analysis of the *cpsF* gene is necessary to accurately determine whether the capsule of an isolate corresponds to serotype 9 or 11. Under these circumstances, the designation of the hybrid serotype 9/11 remains a valid approach [3]. The geographical distribution of such serotypes varies in different countries/continents [1]. Virulence of *A. pleuropneumoniae* isolates belonging to the same serotype, but also within the same serotype, may also vary; some strains can produce high mortality, others are low or non-virulent, and yet others are intermediate in virulence [1]. Depending on the geographical regions, isolates belonging to serotypes 1, 2, 4, 5, 7 and 9/11 are, in general, considered as highly virulent [1]. On the other hand, serotypes 3 and 12 are usually considered as low virulent worldwide [1]. The most important factors involved on the pathogenesis of the infection are the secreted protein RTX-toxins, ApxI, ApxII, and ApxIII [3]. These toxins are coded by the following genes: *apxlCA*, *apxIICA*, *apxIIICA*, *apxIBD* and *apxIIIBD*, where the *CA* genes encode the activator *C* and the structural toxin *A* and the *BD* genes the cis- or trans-acting Type 1 Secretion System. In general, with a few exceptions, most isolates belonging to the same serotype share the same *apx* pattern [4–6].

To enhance the serotyping of *A. pleuropneumoniae* for diagnostic and epidemiological purposes, a multiplex PCR assay has recently been developed for the comprehensive LPS O-antigen typing of all *A. pleuropneumoniae* isolates which would be complementary to that used for routine CPS serotyping [7]. However, due to the high similarity of epitopes and the corresponding genetic sequences among certain serotypes (e.g., 1, 9, and 11; 3, 8, and 15), cross-reactivity can occur [1]. The antigenicity of LPS O-antigens also plays a crucial role in diagnosing silent infections in livestock. Serological testing has been extensively employed for the identification, control, and eradication of virulent serotypes and remains the most effective tool for detecting subclinical *A. pleuropneumoniae* infections [1]. Apart from the assay detecting antibodies against the ApxIV toxin, which does not distinguish between virulent and non-virulent strains, an ELISA test utilizing purified O-chain LPS antigens has been successfully implemented over the past three decades to identify antibodies against specific serotypes or serogroups of *A. pleuropneumoniae*, facilitating the detection of subclinical infections [8].

As mentioned above, serotype 12 is widely distributed in commercial herds which are subclinically infected [9] and is rarely recovered from diseased pigs; the few reports on isolates recovered from ill animals stated isolation rates between 0.2 to 8.4% [4, 5, 10–13]. A recent study reported the isolation of this serotype from tonsils of wild boars [14]. In the present report we characterized four atypical field strains of *A. pleuropneumoniae* serotype 12 isolated from serious pleuropneumonia outbreaks in Chile from 2019 to 2021. These strains were compared with a collection of strains recovered from diseased pigs mostly in Canada and USA and also with atypical strains previously recovered in Japan.

## Materials and methods

### Bacterial strains and growth conditions

The list of field strains used in the current study is presented in Table 1. The Chilean strains (19–073, 21–0011, 21–0012 and 21–001-3) were recovered in four different farms; animals were clinically affected presenting unexpected high morbidity and mortality due to *A. pleuropneumoniae*. All farms were free of Porcine Reproductive and Respiratory Syndrome virus (PRRSv) and vaccinated against *Mycoplasma hyopneumoniae* infection. Two strains originated from two farms that had some epidemiological connexion due to infrequent gilt exchanges in the past, but they were still included in this study individually. The other farms did not have any epidemiological relationship. In addition, a total of 44 strains (24 from Canada, 19 from USA and 1 from France) received by our diagnostic laboratory during the last 20 years were included in the current study. Bacterial strains were cultured in PPLO (Difco Laboratories, Detroit, MI, USA) agar plates supplemented with 0.1% glucose, 5% horse serum and 10% yeast extract as previously described [15].

**Table 1:** *Actinobacillus pleuropneumoniae* strains serotype 12 included in the current study and their gene toxin profile (as determined by PCR).

*Actinobacillus pleuropneumoniae* strains			Gene toxin profile				
Strain number	Year of isolation	Country of origin	*apxICA*	*apxIICA*	*apxIIICA*	*apxIBD*	*apxIIIBD*
A04-0696	2004	Canada	NEG	POS	NEG	POS	NEG
A04-1484	2004	USA	NEG	POS	NEG	POS	NEG
A04-1526	2004	USA	NEG	POS	NEG	POS	NEG
19–045	2019	Canada	NEG	POS	NEG	POS	NEG
19–068	2019	USA	NEG	POS	NEG	POS	NEG
19–014	2018	USA	NEG	POS	NEG	POS	NEG
18–069	2018	USA	NEG	POS	NEG	POS	NEG
18–070	2018	USA	NEG	POS	NEG	POS	NEG
18–053	2018	Canada	NEG	POS	NEG	POS	NEG
18–008	2018	Canada	NEG	POS	NEG	POS	NEG
17–071	2017	USA	NEG	POS	NEG	POS	NEG
17–059-1	2017	Canada	NEG	POS	NEG	POS	NEG
17–059-2	2017	Canada	NEG	POS	NEG	POS	NEG
17–059-3	2017	Canada	NEG	POS	NEG	POS	NEG
17–039	2017	USA	NEG	POS	NEG	POS	NEG
17–030	2017	Canada	NEG	POS	NEG	POS	NEG
17–013	2017	USA	NEG	POS	NEG	POS	NEG
16–043	2016	USA	NEG	POS	NEG	POS	NEG
16–038	2016	Canada	NEG	POS	NEG	POS	NEG
15–071-1	2015	USA	NEG	POS	NEG	POS	NEG
15–071-2	2015	USA	NEG	POS	NEG	POS	NEG
15–014	2015	Canada	NEG	POS	NEG	POS	NEG
15–015	2015	Canada	NEG	POS	NEG	POS	NEG
14–054	2014	Canada	NEG	POS	NEG	POS	NEG
14–032	2014	Canada	NEG	POS	NEG	POS	NEG
14–018	2014	Canada	NEG	POS	NEG	POS	NEG
14–008	2014	USA	NEG	POS	NEG	POS	NEG
13–046-2	2013	USA	NEG	POS	NEG	POS	NEG
13–035	2013	Canada	NEG	POS	NEG	POS	NEG
13–018	2013	Canada	NEG	POS	NEG	POS	NEG
13–008-1	2013	Canada	NEG	POS	NEG	POS	NEG
13–008-2	2013	Canada	NEG	POS	NEG	POS	NEG
13–012	2013	Canada	NEG	POS	NEG	POS	NEG
12–066-2	2012	Canada	NEG	POS	NEG	POS	NEG
12–038	2012	Canada	NEG	POS	NEG	POS	NEG
12–011	2012	USA	NEG	POS	NEG	POS	NEG
9499/84	1984	Canada	NEG	POS	NEG	POS	NEG
8329/85	1985	Denmark	NEG	POS	NEG	POS	NEG
A05-0185–2	2005	France	NEG	POS	NEG	POS	NEG
A05-0565–2	2005	USA	NEG	POS	NEG	POS	NEG
A05-0660–4	2005	USA	NEG	POS	NEG	POS	NEG
A06-0061–1	2006	Canada	NEG	POS	NEG	POS	NEG
16–058	2016	USA	NEG	POS	POS	POS	POS
15–007	2015	Canada	NEG	POS	POS	POS	POS
13–074	2013	USA	NEG	POS	POS	POS	POS
19–073	2019	Chile	NEG	POS	POS	POS	POS
21–001-1	2021	Chile	NEG	POS	POS	POS	POS
21–001-2	2021	Chile	NEG	POS	POS	POS	POS
21–001-3	2021	Chile	NEG	POS	POS	POS	POS

In addition, purified DNA of five additional strains from Japan were included: strains 803, 2680 and 2725 previously identified by using agar gel precipitation test as non-encapsulated serotype K12:O3 [16] as well as encapsulated strains KG925 and KG1616, recovered in 2016 and 2024, respectively, also previously identified in the Japanese laboratory as K12:O3 (Table 1). Except for the Japanese strains, for which the information was already available, the serotype of other strains as well as their toxin genetic pattern were confirmed by multiplex-PCR tests as previously reported [2, 6].

### Whole genome sequencing

The genetic diversity of all strains of *A. pleuropneumoniae* serotype 12 was studied by whole genome sequencing. In addition to serotype 12 strains, the genome sequences of reference strains representing all known *A. pleuropneumoniae* serotypes were also included in the analyses. For each isolate, bacterial colonies were collected from supplemented PPLO agar plates. DNA was extracted using the PureLink™ Genomic DNA Mini Kit (Invitrogen, Carlsbad, CA, USA). Incubation temperatures were increased from the manufacturer’s suggested 55 °C to 62 °C, and 1.4 mm ceramic beads were added to the prepared lysates to increase extracted genomic material. Extracted DNA was quantified using Qubit™ dsDNA Quantification Assay Kit and a Qubit^®^ 3.0 Fluorometer (ThermoFisher Scientific, Waltham, MA, USA). For Oxford Nanopore sequencing, samples were diluted to 50 ng/µL. For the Illumina MiniSeq, samples were diluted to 0.20 ng/µL. Sequencing of isolates on the Nanopore was performed using the Oxford Nanopore Rapid Barcoding Sequencing Kit (Oxford Nanopore Technologies, Oxford, UK) and GridION. During the library preparation, the volume of 10 mM Tris–HCl pH 8.0 with 50 mM NaCl was increased from 10 µL to 15 µL when doing the final wash of the pelleted magnetic beads. The final volume of eluted DNA was increased from 10 µL to 12 µL. Nextera Library preparation (Illumina, San Diego, CA, USA) and paired-end sequencing (2 × 150 bp) was performed using an Illumina MiniSeq according to manufacturer’s protocol.

Illumina and Nanopore reads were filtered using fastp version 0.23.4 [17] and filtlong version 0.2.1, respectively. Subsequently, Unicycler version 0.5.0 [18] was used for hybrid assembly. All genomic sequences have been deposited in the public GenBank database (Additional file 1; information to come from GeneBank). Bakta version 1.8.2 was used for annotation [19] and EasyFig version 2.2.2 [20] was employed to compare the capsule and LPS loci. A molecular phylogeny based on the softcore genome (genes present in over 95% of the dataset) was constructed as previously described [21]. The homology search for Apx toxins was performed using DIAMOND version [22].

### Virulence studies

To evaluate the virulence of a representative atypical *A. pleuropneumoniae* strain, an experimental infection was carried out. The atypical Chilean representative strain 21–001-1 as well as the reference *A. pleuropneumoniae* serotype 12 strain 8329/85 were used to experimentally infect animals. Bacterial inoculum was prepared from freshly streaked supplemented PPLO as mentioned above. After approximately 5 h at 37 °C and 5% CO_2_, bacterial growth was resuspended in commercial phosphate buffer saline (PBS) and adjusted to a concentration of approximately 2 × 10^7^ CFU/mL. Bacterial concentration was confirmed by dilution of inoculum and plating on supplemented PPLO. The animal study was carried out in accordance with the recommendations of the guidelines and policies of the Canadian Council on Animal Care and the principles outlined in the Guide for the Care and Use of Laboratory Animals.

Thirty-two 6-week-old conventional male or female piglets were included in the study. The animals came from a farm free from *A. pleuropneumoniae*, *M. hyopneumoniae* and PRRSv. Once at facilities, animals were weighed and distributed into three groups based on their bodyweight and sexes. After one week of acclimation, two groups of twelve pigs were intratracheally challenged with a mean dose of 8 × 10^7^ CFU of either strain 21–001-1 or 8329/85 in 5 mL of PBS. The remaining 8 pigs were similarly inoculated with PBS. Control and inoculated animals were housed in three different isolated level II pens. Animals were weighed and bled at challenge and necropsy days. Following the challenge, pigs were monitored three times/day over a period of nine days for the presence of clinical signs and mortality. Animals reaching the end-point limit accepted by the ethical committee were humanely euthanized as previously described [23]. The calculation of an individual clinical scores was based on daily examinations, following the method described by Sibila et al. [24], with some modifications. Overall, the clinical score was composed by eight different individual parameters: breathing noise, type of respiration, breathing rate, coughing, skin colour, posture, behaviour, body temperature, and nasal secretion [24]. Each parameter without pathological expression was rated 0, the highest degree was given a value of 4. If an animal dies or was euthanized because of the infection, 5 points were awarded to that animal for each other day of the trial period. Cumulative clinical scores were calculated for each pig as a sum of daily clinical scores.

Animal weight was also recorded on challenge and necropsy days. Average daily gain (ADG) was calculated as the weight at necropsy minus the weight at challenge divided by the days lapsed between them [24]. A post-mortem examination procedure was conducted for all pigs. Lungs were evaluated for weight/body weight ratio (LW/BW). This method allows the quantification of the increased lung weight due to lesions with respect to the whole weight of the pig. This ratio was calculated using the following formula: (Weight of the lung/body weight) × 100 [24]. Lung samples were also taken for bacterial isolation, serotyping and histopathology studies from all euthanized animals as well as survival animals at the end of the trial. For histopathology studies, slides were analysed blindly by a board-certified veterinary pathologist (G. St-Jean). The presence or absence of lesions was blindly determined in each sample and the distribution of lesions reported. Samples were collected following a standardized procedure (right and left caudal, middle and anteroventral regions, tracheobronchial lymph nodes) and specific lesions noted macroscopically. Samples were fixed in 10% buffered formalin, embedded in paraffin, and sectioned at a thickness of 3 µm. The slides were stained with Hematoxyline Phloxyn Saffron (HEPS). The severity of characteristic lesions of *A. pleuropneumoniae*, including inflammation, hemorrhage, fibrin exudation and necrosis were scored as 0 (no), 1 (minimal), 2 (mild), 3 (moderate), 4 (marked) or 5 (severe), and the score recorded based on the more severely affected lobes or submitted lesions. Finally, tonsil samples were tested by PCR for *A. pleuropneumoniae* serotype 12 [2, 4] and serum samples were taken to test for the presence of antibodies using long-chain LPS-ELISA tests [15].

### Statistics

Normality of data was verified using the Shapiro–Wilk test. Accordingly, parametric (unpaired t-test) or non-parametric tests (Mann–Whitney rank sum test), where appropriate, were performed to evaluate statistical differences between groups. *p* < 0.05 was considered as statistically significant.

## Results

### Characterization of strains

All tested strains were confirmed to be serotype 12 by multiplex PCR [2]. However, the toxin genotype profile of some strains was atypical based on PCR results (Table 1) and confirmed based on sequence homology (Additional file 2). Indeed, the gene toxin profile usually reported for this serotype is *apxIICA* and *apxIBD*, suggesting the production and secretion of ApxII only [5]. Nonetheless, the four Chilean strains possess in addition *apxIIICA*, and *apxIIIBD*, like what was reported for the atypical Japanese strains [16, 25], suggesting the production and secretion of ApxII and ApxIII, like strains belonging to serotypes 2, 6, 8 and 15 [5]. Since the original profile of serotype 12 was done in a reference strain and a few field strains many years ago [26], we decided to test the toxin genotype of field isolates we had in our laboratory. Of the 44 strains, 41 presented an expected profile of typical serotype 12 strains and three presented an atypical profile, identical to the Chilean isolates. These three strains were 16–058 and 13–074 (from USA) and 15–007 (from Canada) (Table 1).

### Comparative genomic analysis

**Figure 1:**
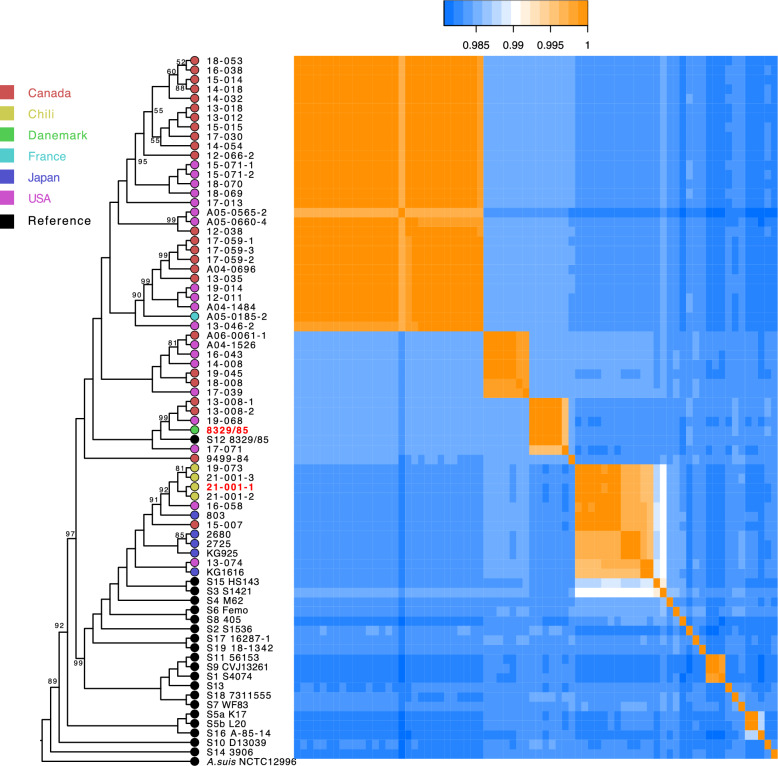
**Molecular phylogeny of the strains under study, as well as strains representing known serotypes of *Actinobacillus pleuropneumoniae*.** Colored circles represent the geographic origin of strain isolation for the bacteria sequenced for the present study. Bootstrap values below 100 are indicated for the various nodes of the tree. The heatmap shows Average Nucleotide Identity (ANI) values between genomic sequences. *Actinobacillus suis* NCTC12996 (GenBank LT906456.1) was used as an outgroup to root the tree.

**Figure 2:**
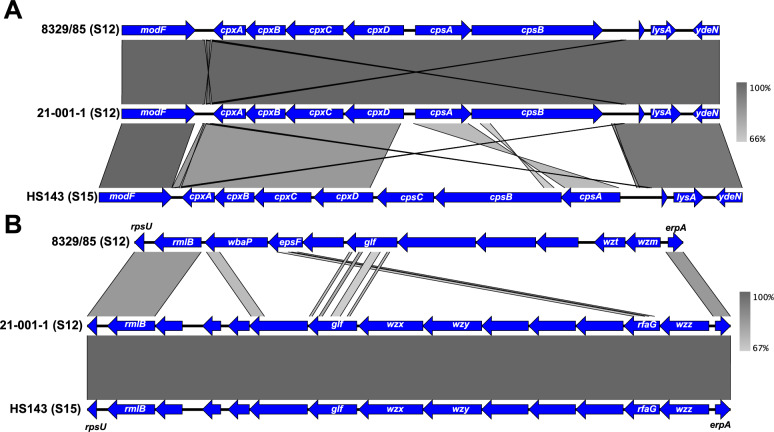
**Alignments of the CPS loci (A) and LPS loci (B) for strains 8329/85 (reference strain serotype 12), 21–001-1 (atypical strain serotype 12), and HS143 (reference strain serotype 15) of *Actinobacillus pleuropneumoniae*.** Homologous regions between sequences are shown in grey. The genes present are represented by the blue arrows.

A total of 1799 orthologous gene sequences were used for phylogenetic reconstruction. The corresponding accession numbers (BioSample) are presented in Additional file 1. The resulting tree indicates that strains identified as serotype 12 are paraphyletic, comprising a large group that includes most strains as well as the reference strain 8329/85, and a smaller, distinct group (Figure 1). Within each of the two groups, strains tend to form additional subgroups based on the geographical origin of their isolation. The smaller group consists of atypical strains from Chile, Japan, the two atypical strains from the USA (16–058, 13–074), and that from Canada (78–15-007), demonstrating a broad geographical distribution. A matrix of Average Nucleotide Identity (ANIm) values reveals that these atypical strains share greater genomic sequence identity with the serotype 15 reference strain (HS143) than with other serotype 12 strains. Alignment of the loci encoding the capsule and LPS genes in the reference strains of serotypes 12 and 15, as well as in the atypical Chilean strain 21–001-1, clearly shows that strain 21–001-1 possesses the capsule genes of serotype 12, while its LPS genes correspond to those of serotype 15 (Figure 2).

Analysis of the structural ApxIIIA toxin revealed that the atypical strains from Chile, Japan and the two from USA had an ApxIII subtype similar as found in serotypes 3, 4, 6, 8 and 15, which show a significant difference in the C-terminal half of the molecule (unpublished data). It is also worth noting that the *apxIV* gene exhibits substantial sequence variability (Additional file 2), as initially found by Scheller et al. [27] and recently documented for other *A. pleuropneumoniae* strains [28].

### Virulence studies

**Figure 3:**
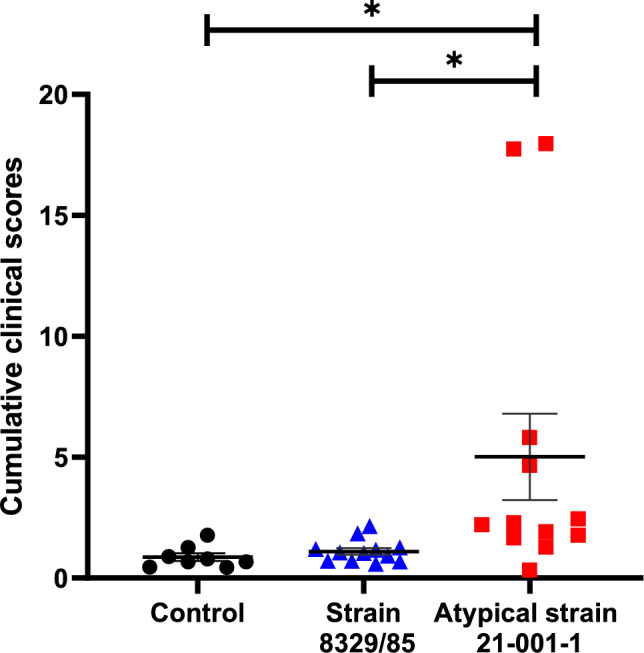
**Cumulative clinical scores of animals infected with either *Actinobacillus pleuropneum*oniae serotype 12 reference strain or the atypical strain 21–001-1**. *Indicates a significant difference (*p* < 0.05).

**Figure 4:**
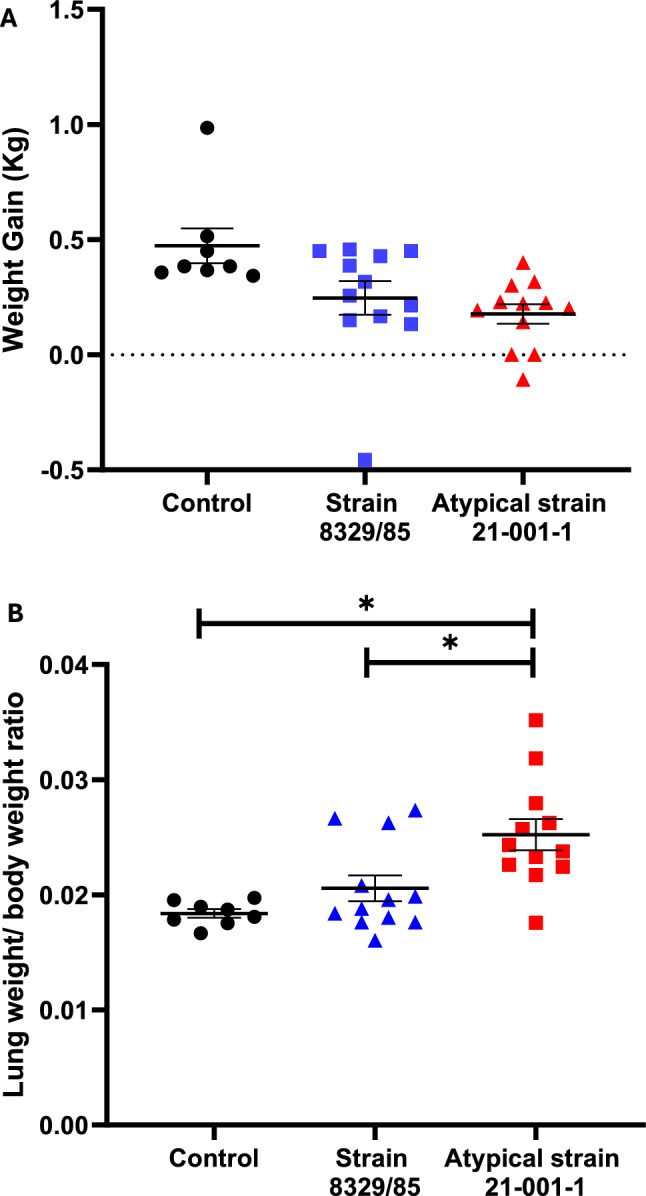
Mean average daily weigh gain (ADG) (Kg) (**A**) and lung weight/body weight ratio of animals (B) experimentally infected with either the reference (8329/85) or atypical (21–001-1) strain of *Actinobacillus pleuropneumoniae* serotype 12.

Animals from the control group remained healthy throughout the study. Two animals infected with the atypical strain 21–001-1 were euthanized 24 h-post infection as they presented severe respiratory problems, depression and blood in nasal secretions. The mean of cumulative clinical score was significantly higher (*p* < 0.05) for animals infected with the atypical strain 21–001-1 when compared to those infected with the reference strain (Figure 3). Interestingly, no significant differences were observed between the control group and animals infected with the reference strain (*p* > 0.05). The ADG of animals infected with the atypical strain was significantly different from the control group (*p* < 0.01) but similar to animals infected with the reference strain. The latter group did not present an ADG different from the control group (*p* > 0.05) (Figure 4A). The ratio lung/body weight was also higher in animals infected with the atypical 21–001-1 strain when compared to those infected with the traditional strain (*p* < 0.01) and the control group (*p* < 0.05) (Figure 4B). No statistical differences were observed between the ratio observed for animals infected with the traditional strain and the control group (*p* > 0.05).

**Figure 5:**
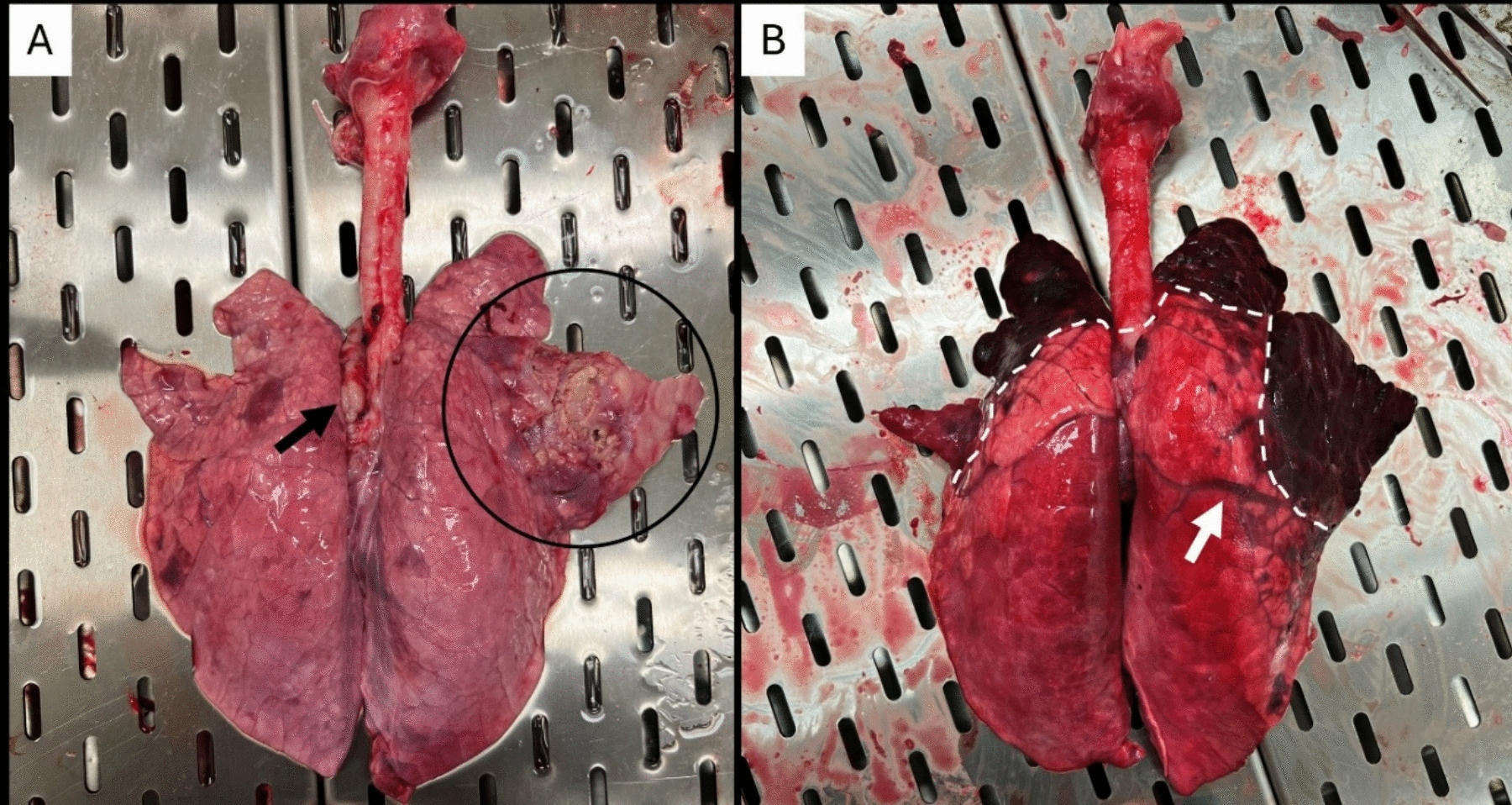
**Gross lungs findings observed following inoculation with atypical strain 21–001-1 of *Actinobacillus pleuropneumoniae* serotype 12 in pigs**. There was interindividual variations of severity and type of lesions throughout the experiment. **A.** Mild severity, characterized by anteroventral bronchopneumonia with abscess formation (circled in black), associated with lymphadenomegaly of the tracheobronchial lymph nodes. **B.** Marked severity, characterized by severe anteroventral necrohemorragic pneumonia (delineated by white dashed line). The remaining parenchyma is uncollapsed, congested and edematous (interlobular edema-white arrow). Bacterial culture was positive for *A. pleuropneumoniae* in lungs for both cases.

**Figure 6:**
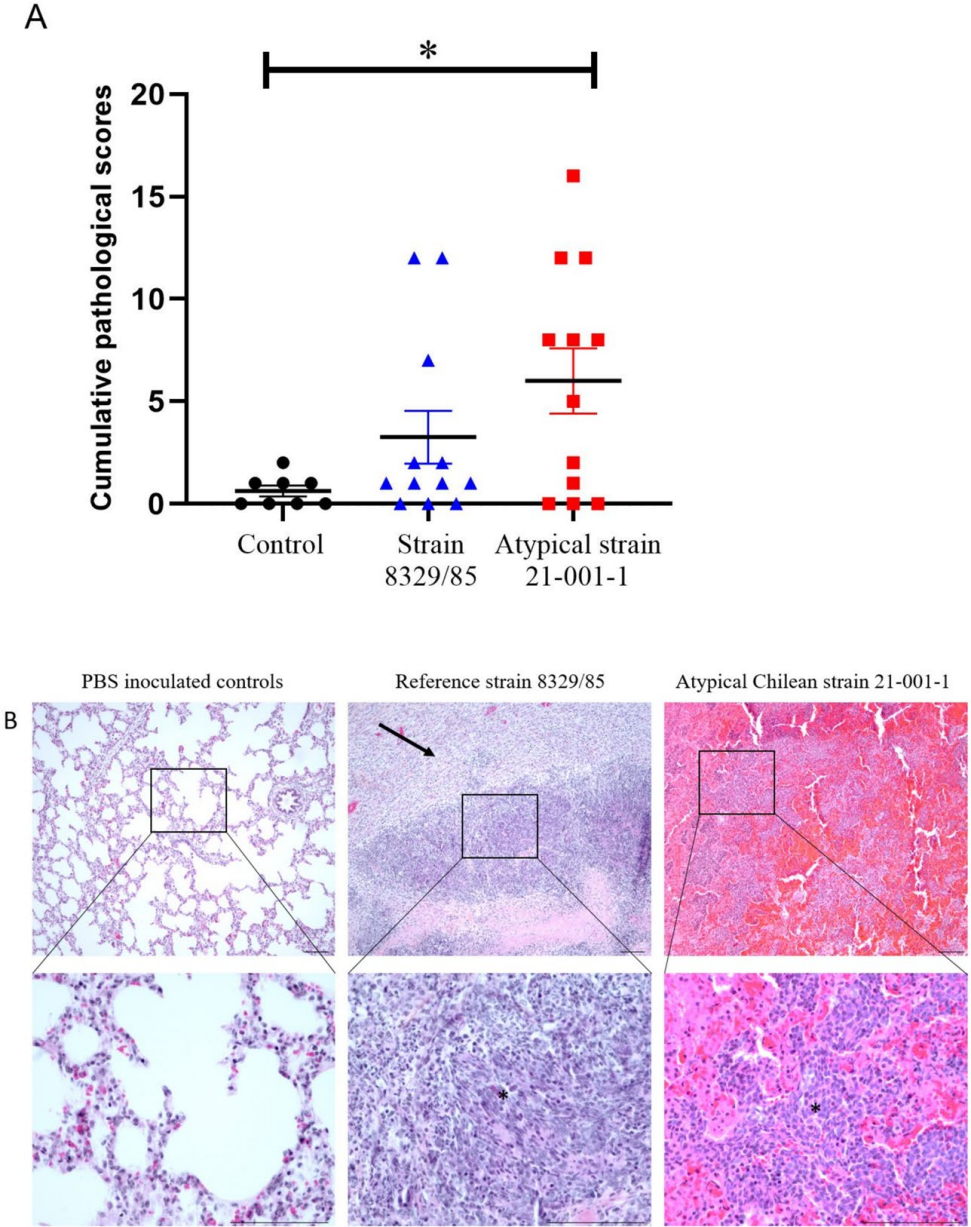
**Histopathological evaluation of pigs inoculated with PBS, reference strain 8329/85 and atypical strain 21–001-1**. **A** Total pathological scores (inflammation, hemorrhages, fibrin exudation and necrosis) recorded for the different strains and PBS controls, data are presented as mean_ ± SEM, *p < 0.05. **B** Microscopic features of each group, with noticeable hemorrhages in the 21–001-1 strain. Chronic changes, such as granulation tissue (black arrow), are noted in the 8329/85 strain. Typical “oat cells” (*) can be noted in both groups, although more apparent in the reference strain 8329/85. Images on the second row represent higher magnification of the image above (square). HPS stain, bar = 100 µm.

Typical lesions of pleuropneumonia were observed in some animals infected with the atypical strain 21–001-1, although there was interindividual variations of severity and type of lesions throughout the experiment (Figures 5A and B). In histopathology, although there was no statistical difference between the pathological scores of the reference strain 8329/85 and atypical strain 21–001-1, there was a clear trend suggesting increased severity of the lesions. This trend was further exemplified by the significant statistical difference (*p* < 0.05) observed between the atypical strain 21–001-1 and the PBS controls, a difference not observed between samples from animals infected with the reference strain 8329/85 and the controls (Figure 6A). The distribution of the lesions in the lungs was often wider in the atypical strain compared to the reference strain. The most distinctive microscopic feature of the atypical strain 21–001-1 was consistent with acute inflammatory changes, including severe hemorrhages and fibrin exudation, compared to the reference strain 8329/85 in which the more severe lesions often had chronic changes, such as the development of granulation, with a lower prevalence and severity of hemorrhages and/or fibrin exudation (Figure 6B). These observations would explain the more serious clinical course of the disease observed with latter strain.

Finally, no *A. pleuropneumoni*ae was detected neither in tonsils nor in lungs of the control group, as expected. A higher number of pigs was positive by culture of lungs of animals infected by the atypical strain (67%), when compared to those infected with the reference strain (33%). However, more positive samples (75% vs 50%) were detected by PCR in tonsils of animals infected with the reference strain. Concerning the serological response, and due to the relatively short time the animals were kept for the experiment (10 days after infection), a low serological response was expected. Serological results showed that control animals remained negative (optical densities or O.D._414_ < 0.1) for all serotypes of *A. pleuropneumoniae* at the end of the trial. Although animals were infected for less than 10 days, three animals from the challenged group with the reference strain presented O.D._414_ higher than 0.2 and two animals presented values between 0.1 and 0.2. with an ELISA test based on O-chain LPS of serotype 12. Animals infected with the atypical strain 21–001-1 unexpectedly remained completely negative for serotype 12, but 8 animals presented clear positive results (O.D._414_ higher than 0.2) for O-chain LPS of *A. pleuropneumonaie* serotypes 3/6/8/15/17 (Additional file 3).

## Discussion

Porcine pleuropneumonia, caused by the bacterial pathogen *A. pleuropneumoniae*, leads to high economic losses in affected swine herds in most countries of the world. It has been largely reported that virulence of isolates is normally correlated with the serotype involved with different geographical distribution [1].

Although this statement is still true, the pattern of the infection is somehow changing. For example, in the past, serious outbreaks of swine pleuropneumonia were uncommon in North America [1]. However, two serious outbreaks caused by non-traditional highly virulent serotypes (8 and 15) caused serious losses in recent years in USA [29, 30]. Another serious outbreak affecting several unrelated farms also caused by a serotype 8 occurred in Canada in 2024 (M. Gottschalk, unpublished data). On the other hand, certain serotypes have been always considered low virulent, such as serotypes 3 and 12 [1]. For these reasons, the accurate identification and characterization of *A. pleuropneumoniae* isolates is essential for diagnosis, control, and surveillance of porcine pleuropneumonia [1]. In the current study we characterized serotype 12 strains recovered from severe cases of pleuropneumonia in Chile.

Apx toxins belonging to the RTX toxin family are considered as major virulence factors of *A. pleuropneumoniae* [31]. In general, with some exceptions, field *A. pleuropneumoniae* strains present a toxin profile identical to that described for the respective serotypes [26, 28, 32, 33]. When atypical patterns are observed, in most cases, there is a loss rather than an acquisition of genes [5, 34]. For serotype 2 North American strains lacking genes for the production of ApxIII (with a reduced virulence potential) have been described [1]. The atypical strains characterized in the current study possess a toxin gene profile including *apxIIICA* and *apxIIIBD*, indicating the capacity to produce ApxIII in addition to ApxII, which is not common for serotype 12 strains. Indeed, the latter typically carry only *apxIICA* and *apxIBD*. It is important to notice that the ApxIII toxin found in these atypical strains secrete the subtype of ApxIII typically found in serotypes 3, 4, 6, 8 and 15, which showed to have a significant impact in vaccines against *A. pleuropneumoniae* serotype 15 outbreaks in Australia [35]. This differentiation has important implications in the choice of the vaccine used for protecting the pigs in respective outbreaks. Indeed, in general, the pattern observed with the Chilean strains is more in line with higher virulent serotypes such as 2, 6, 8, 15 and 17 [26]. Interestingly, similar observations were previously described with encapsulated and non-encapsulated Japanese serotype 12 strains [16, 25]. In the current study, a similar pattern was observed with Japanese strains and three North American strains, while all others presented a typical serotype 12 *apx* profile.

Comparative genomic analysis confirmed that while all atypical strains included in the current study retained the capsule gene cluster of serotype 12, their O-LPS biosynthesis loci were closely aligned with those of serotype 15. Indeed, these strains are more closely related to the reference strain of *A. pleuropneumoniae* serotype 15 rather than that of serotype 12. The K12:O15 hybrid genotype was consistently found in all atypical strains included in this study from different geographical origin (Chile, USA, Canada and Japan). Whole genome sequencing revealed all these atypical strains clustered together and separately from the classical serotype 12 group, which suggests a possibly global distribution of this clone. Discrepancies between CPS serotype and LPS O-antigen profiles have been previously reported in the literature, raising concerns about the genomic plasticity of the organism and its implications for diagnostics. Previous studies have identified isolates carrying CPS genes of one serotype but expressing LPS O-antigen structures typical of another, such as strains of K2:O7, K19:O4, K19:O3, K1:O7, K13:O10 and K4b:O3 [7, 36–39]. As mentioned, *A. pleuropneumoniae* K12:O3 have also been previously described in Japan [16, 25]. These observations suggest that recombination events affecting capsule or LPS biosynthesis loci may not be rare and could lead to misclassification or underestimation of strain virulence when diagnostics rely solely on conventional serotyping.

Another important diagnostic consideration arises from serological testing. The atypical strains triggered an antibody response in infected animals that was detected by an ELISA using O-LPS antigens of serotypes 3/6/8/15/17, but not of that of serotype 12. This mismatch further supports the presence of serotype 15-like LPS structures and demonstrates how reliance on serotype-specific serology could lead to false-negative results in infected animals. Indeed, in field settings, this may hinder the identification of outbreaks and compromise control measures. Given the increasingly documented cases of strains with discordant CPS and LPS profiles, the integration of both capsule and LPS typing into diagnostic workflows is warranted. The use of multiplex PCR assays targeting both loci, combined with whole-genome sequencing for comprehensive characterization when needed, should be considered best practice for reference laboratories.

Finally, the in vivo experimental infection results provided compelling evidence for the enhanced virulence of the atypical strain 21–001-1. Pigs infected with this strain showed significantly higher clinical scores, more extensive lung lesions, and increased lung-to-body weight ratios compared to those infected with the reference strain 8329/85. The presence of acute lesions characterized by hemorrhages and fibrin exudation further underscores the aggressive nature of the atypical strain, contrasting with the low chronic inflammatory features observed with the reference strain. These results are particularly notable as animals infected with the reference strain showed a clinical and pathological profile comparable to the PBS control group, reinforcing the traditionally held view of serotype 12 as having low virulence. A relative high variability of clinical signs among animals infected with the atypical strains was observed. This was expected, as this strain possesses an intermediate virulence potential. When highly virulent strains are used (such as serotype 5 strains), most animals will be severely affected [40].

In conclusion, our study identifies and characterizes a distinct group of *A. pleuropneumoniae* strains with serotype 12 capsule genes, serotype 15 LPS genes, and enhanced virulence. Their detection across multiple continents suggests that such strains may be more widespread than previously recognized and that their clinical significance has likely been underestimated. CPS-based PCR may still be considered as a first step routine serotyping method as most strains will present typical CPS/LPS combinations. However, in cases when serotyping results do not correlate with serological results (a positive serotype present in a herd with negative serological results for such serotype), the LPS-based PCR can be used to detect the presence of heterologous CPS/LPS serotypes. Finally, the results of the current study suggest careful re-evaluation of serotype-based classifications in light of evolving genomic evidence.

## Supplementary Information

**Additional file 1. Accession numbers (BioSample) of the *Actinobacillus pleuropneumoniae* strains studied.**

**Additional file 2.** **Presence of *apx* genes (data from whole genome sequencing) in the studied *Actinobacillus pleuropneumoniae* strains.**

**Additional file 3.* Actinobacillus pleuropneumonie* LC-LPS ELISA serology results at euthanasia.** Values are expressed as optical density values (414_ nm_).
