# Correction: Atypical *Actinobacillus pleuropneumoniae* serotype 12 strains with a higher virulence potential

**DOI:** 10.1186/s13567-025-01597-7

**Published:** 2025-07-30

**Authors:** Antony T. Vincent, Sonia Lacouture, Guillaume St-Jean, Rodrigo Tapia, Servane Payen, Michiha Kon, Joachim Frey, Ho To, Marcelo Gottschalk

**Affiliations:** 1 Research Group On Infectious Diseases in Production Animals (GREMIP) and Swine and Poultry Infectious Diseases Research Center (CRIPA), Department of Pathology and Microbiology, Faculty of Veterinary Medicine, University of Montreal, Saint-Hyacinthe, QC J2S 2M2 Canada; 2 Virbac Chile, Cerrillos, Santiago, Chile; 3 Université Laval, Québec, QC Canada; 4 University of Bern, Bern, Switzerland; 5 Nippon Institute for Biological Science, Tokyo, Japan; 6 Faculty of Agriculture and Aquaculture, University of Cuu Long, Vinh Long, Vietnam

**Correction: Veterinary Research (2025) 56:149** 10.1186/s13567-025-01579-9

Following publication of the original article [1], the authors reported that the names of the authors Michiha Kon and Ho To were erroneously transposed.

The incorrect author names are: Kon Michiha and To Ho

The correct author names are: Michiha Kon and Ho To

The original article has been corrected.
