# Supplementary material for: Atypical Actinobacillus pleuropneumoniae serotype 12 strains with a higher virulence potential
Source: Vet Res. 2025 Jul 13;56:149. doi: 10.1186/s13567-025-01579-9 (PMC12255999; doi:10.1186/s13567-025-01579-9)
Supplement: Supplementary file 1 — Additional file 1. Accession numbers (BioSample) of the Actinobacillus pleuropneumoniae strains studied. [file 13567_2025_1579_MOESM1_ESM.docx]

**Additional file 1: Accession numbers (BioSample) of the *Actinobacillus pleuropneumoniae* strains studied.**

| BioSample ID | Strain | Collection Date | Location |
| --- | --- | --- | --- |
| SAMN48231401 | KG925 | 2016 | Japan |
| SAMN48231400 | KG1616 | 2024 | Japan |
| SAMN48231399 | A05-0185-2 | 2005 | France |
| SAMN48231398 | 21-001-3 | 2021 | Chile |
| SAMN48231397 | 21-001-2 | 2021 | Chile |
| SAMN48231396 | 21-001-1 | 2021 | Chile |
| SAMN48231395 | 8329/85 | 1985 | Denmark |
| SAMN48231394 | 9499/84 | 1984 | Canada |
| SAMN48231393 | 19-073 | 2019 | Chile |
| SAMN48231392 | 12-011 | 2012 | USA |
| SAMN48231391 | 12-038 | 2013 | Canada |
| SAMN48231390 | 12-066-2 | 2013 | Canada |
| SAMN48231389 | 13-012 | 2013 | Canada |
| SAMN48231388 | 13-008-2 | 2013 | Canada |
| SAMN48231387 | 13-008-1 | 2013 | Canada |
| SAMN48231386 | 13-018 | 2013 | Canada |
| SAMN48231385 | 13-035 | 2013 | Canada |
| SAMN48231384 | 13-046-2 | 2013 | USA |
| SAMN48231383 | 13-074 | 2013 | USA |
| SAMN48231382 | 14-008 | 2014 | USA |
| SAMN48231381 | 14-018 | 2014 | Canada |
| SAMN48231380 | 803 | 2015 | Japan |
| SAMN48231379 | 14-032 | 2014 | Canada |
| SAMN48231378 | 14-054 | 2014 | Canada |
| SAMN48231377 | 15-007 | 2015 | Canada |
| SAMN48231376 | 15-015 | 2015 | Canada |
| SAMN48231375 | 15-014 | 2015 | Canada |
| SAMN48231374 | 15-071-2 | 2015 | USA |
| SAMN48231373 | 15-071-1 | 2015 | USA |
| SAMN48231372 | 16-038 | 2016 | Canada |
| SAMN48231371 | 16-043 | 2016 | USA |
| SAMN48231370 | 16-058 | 2016 | USA |
| SAMN48231369 | 17-013 | 2017 | USA |
| SAMN48231368 | 17-030 | 2017 | Canada |
| SAMN48231367 | 17-039 | 2017 | USA |
| SAMN48231366 | 17-059-3 | 2017 | Canada |
| SAMN48231365 | 17-059-2 | 2017 | Canada |
| SAMN48231364 | 17-059-1 | 2017 | Canada |
| SAMN48231363 | 17-071 | 2017 | USA |
| SAMN48231362 | 18-008 | 2018 | Canada |
| SAMN48231361 | 18-053 | 2018 | Canada |
| SAMN48231360 | 18-070 | 2018 | USA |
| SAMN48231359 | 18-069 | 2018 | USA |
| SAMN48231358 | 19-014 | 2018 | USA |
| SAMN48231357 | 19-068 | 2019 | USA |
| SAMN48231356 | 19-045 | 2019 | Canada |
| SAMN48231355 | A04-1526 | 2004 | USA |
| SAMN48231354 | A04-1484 | 2004 | USA |
| SAMN48231353 | A04-0696 | 2004 | Canada |
| SAMN48231352 | 2725 | 2017 | Japan |
| SAMN48231351 | 2680 | 2016 | Japan |
| SAMN48231350 | A06-0061-1 | 2006 | Canada |
| SAMN48231349 | A05-0660-4 | 2005 | USA |
| SAMN48231348 | A05-0565-2 | 2005 | USA |
