# Supplementary material for: Atypical Actinobacillus pleuropneumoniae serotype 12 strains with a higher virulence potential
Source: Vet Res. 2025 Jul 13;56:149. doi: 10.1186/s13567-025-01579-9 (PMC12255999; doi:10.1186/s13567-025-01579-9)
Supplement: Supplementary file 2 — Additional file 2. Presence of apx genes (data from whole genome sequencing) in the studied Actinobacillus pleuropneumoniae strains. [file 13567_2025_1579_MOESM2_ESM.docx]

**Additional file 2: Presence of *apx* genes (data from whole genome sequencing) in the studied *Actinobacillus pleuropneumoniae* strains.**

| **Strain** | **Origin** | ***apxIA*** | ***apxIB*** | ***apxIC*** | ***apxID*** | ***apxIIA*** | ***apxIIC*** | ***apxIIIA*** | ***apxIIIB*** | ***apxIIIC*** | ***apxIIID*** | ***apxIVA*** |
| --- | --- | --- | --- | --- | --- | --- | --- | --- | --- | --- | --- | --- |
| 18-053 | Canada | **57,8** | 100 | **74,2** | 99,2 | 100 | 100 | **60,6** | 93,6 | 79,0 | 89,1 | 87,7 |
| 16-038 | Canada | **57,8** | 100 | **74,2** | 99,2 | 100 | 100 | **60,6** | 93,6 | 79,0 | 89,1 | 90,0 |
| 15-014 | Canada | **57,8** | 100 | **74,2** | 99,2 | 100 | 100 | **60,6** | 93,6 | 79,0 | 89,1 | 90,0 |
| 14-018 | Canada | **57,8** | 100 | **74,2** | 99,2 | 100 | 100 | **60,6** | 93,6 | 79,0 | 89,1 | 90,2 |
| 14-032 | Canada | **57,8** | 100 | **74,2** | 99,2 | 100 | 100 | **60,6** | 93,6 | 79,0 | 89,1 | 90,2 |
| 13-018 | Canada | **57,8** | 100 | **74,2** | 99,2 | 100 | 100 | **60,6** | 93,6 | 79,0 | 89,1 | 90,0 |
| 13-012 | Canada | **57,8** | 100 | **74,2** | 99,2 | 100 | 100 | **60,6** | 93,6 | 79,0 | 89,1 | 90,2 |
| 15-015 | Canada | **57,8** | 100 | **74,2** | 99,2 | 100 | 100 | **60,6** | 93,6 | 79,0 | 89,1 | 90,0 |
| 17-030 | Canada | **57,8** | 100 | **74,2** | 99,2 | 100 | 100 | **60,6** | 93,6 | 79,0 | 89,1 | 90,2 |
| 14-054 | Canada | **57,8** | 100 | **74,2** | 99,2 | 100 | 100 | **60,6** | 93,6 | 79,0 | 89,1 | 90,0 |
| 12-066-2 | Canada | **57,8** | 100 | **74,2** | 99,2 | 100 | 100 | **60,6** | 93,6 | 79,0 | 89,1 | 90,0 |
| 15-071-1 | USA | **57,8** | 100 | **74,2** | 99,2 | 100 | 100 | **60,6** | 93,6 | 79,0 | 89,1 | 97,4 |
| 15-071-2 | USA | **57,8** | 100 | **74,2** | 99,2 | 100 | 100 | **60,6** | 93,6 | 79,0 | 89,1 | 90,2 |
| 18-070 | USA | **57,8** | 100 | **74,2** | 99,2 | 100 | 100 | **60,6** | 93,6 | 79,0 | 89,1 | 90,2 |
| 18-069 | USA | **57,8** | 100 | **74,2** | 99,2 | 100 | 100 | **60,6** | 93,6 | 79,0 | 89,1 | 90,0 |
| 17-013 | USA | **57,8** | 100 | **74,2** | 99,2 | 100 | 100 | **60,6** | 93,6 | 79,0 | 89,1 | 90,2 |
| A05-0565-2 | USA | **57,8** | 100 | **74,2** | 99,2 | 100 | 100 | **60,6** | 93,6 | 79,0 | 89,1 | 98,0 |
| A05-0660-4 | USA | **57,8** | 100 | **74,2** | 99,2 | 100 | 100 | **60,6** | 93,6 | 79,0 | 89,1 | 90,0 |
| 12-038 | Canada | **57,8** | 100 | **74,2** | 99,2 | 100 | 100 | **60,6** | 93,6 | 79,0 | 89,1 | 92,0 |
| 17-059-1 | Canada | **57,8** | 100 | **74,2** | 99,2 | 100 | 100 | **60,6** | 93,6 | 79,0 | 89,1 | 98,0 |
| 17-059-3 | Canada | **57,8** | 100 | **74,2** | 99,2 | 100 | 100 | **60,6** | 93,6 | 79,0 | 89,1 | 98,0 |
| 17-059-2 | Canada | **57,8** | 100 | **74,2** | 99,2 | 100 | 100 | **60,6** | 93,6 | 79,0 | 89,1 | 98,0 |
| A04-0696 | Canada | **57,8** | 100 | **74,2** | 99,2 | 100 | 100 | **60,6** | 93,6 | 79,0 | 89,1 | 98,0 |
| 13-035 | Canada | **57,8** | 100 | **74,2** | 99,2 | 100 | 100 | **60,6** | 93,6 | 79,0 | 89,1 | 98,1 |
| 19-014 | USA | **57,8** | 100 | **74,2** | 99,2 | 100 | 100 | **60,6** | 93,6 | 79,0 | 89,1 | 98,1 |
| 12-011 | USA | **57,8** | 100 | **74,2** | 99,2 | 100 | 100 | **60,6** | 93,6 | 79,0 | 89,1 | 98,0 |
| A04-1484 | USA | **57,8** | 100 | **74,2** | 99,2 | 100 | 100 | **60,6** | 93,6 | 79,0 | 89,1 | 98,1 |
| A05-0185-2 | France | **57,8** | 100 | **74,2** | 99,2 | 100 | 100 | **60,6** | 93,6 | 79,0 | 89,1 | 98,0 |
| 13-046-2 | USA | **57,8** | 100 | **74,2** | 99,2 | 100 | 100 | **60,6** | 93,6 | 79,0 | 89,1 | 98,0 |
| A06-0061-1 | Canada | **57,8** | 100 | **74,2** | 99,2 | 100 | 100 | **60,6** | 93,6 | 79,0 | 89,1 | 98,9 |
| A04-1526 | USA | **57,8** | 100 | **74,2** | 99,2 | 100 | 100 | **60,6** | 93,6 | 79,0 | 89,1 | 98,9 |
| 16-043 | USA | **57,8** | 100 | **74,2** | 99,2 | 100 | 100 | **60,6** | 93,6 | 79,0 | 89,1 | 98,9 |
| 14-008 | USA | **57,8** | 100 | **74,2** | 99,2 | 100 | 100 | **60,6** | 93,6 | 79,0 | 89,1 | 98,9 |
| 19-045 | Canada | **57,8** | 100 | **74,2** | 99,2 | 100 | 100 | **60,6** | 93,6 | 79,0 | 89,1 | 98,2 |
| 18-008 | Canada | **57,8** | 100 | **74,2** | 99,2 | 100 | 100 | **60,6** | 93,6 | 79,0 | 89,1 | 98,1 |
| 17-039 | USA | **57,8** | 100 | **74,2** | 99,2 | 100 | 100 | **60,6** | 93,6 | 79,0 | 89,1 | 98,3 |
| 13-008-1 | Canada | **57,8** | 99,9 | **74,2** | 99,2 | 100 | 100 | **60,6** | 93,5 | 79,0 | 89,1 | 98,8 |
| 13-008-2 | Canada | **57,8** | 99,9 | **74,2** | 99,2 | 100 | 100 | **60,6** | 93,5 | 79,0 | 89,1 | 98,8 |
| 19-068 | USA | **57,8** | 99,9 | **74,2** | 99,2 | 100 | 100 | **60,6** | 93,5 | 79,0 | 89,1 | 98,8 |
| 8329/85 | Danemark | **57,8** | 99,9 | **74,2** | 99,2 | 100 | 100 | **60,6** | 93,5 | 79,0 | 89,1 | 98,8 |
| 17-071 | USA | **57,8** | 99,9 | **74,2** | 99,2 | 100 | 100 | **60,6** | 93,5 | 79,0 | 89,1 | 98,8 |
| 9499/84 | Canada | **57,8** | 100 | **74,2** | 99,2 | 100 | 100 | **60,6** | 93,6 | 79,0 | 89,1 | 90,9 |
| 19-073 | Chile | **67,3** | 100 | **74,0** | 99,2 | 100 | 100 | 100 | 99,2 | 100 | 99,4 | 99,4 |
| 21-001-3 | Chile | **67,3** | 100 | **74,0** | 99,2 | 100 | 100 | 100 | 99,2 | 100 | 99,4 | 96,8 |
| 21-001-1 | Chile | **67,3** | 100 | **74,0** | 99,2 | 100 | 100 | 100 | 99,2 | 100 | 99,4 | 96,8 |
| 21-001-2 | Chile | **67,3** | 100 | **74,0** | 99,2 | 100 | 100 | 100 | 99,2 | 100 | 99,4 | 96,8 |
| 16-058 | USA | **67,3** | 100 | **74,0** | 99,2 | 100 | 100 | 100 | 99,2 | 100 | 99,4 | 98,2 |
| 803 | Japan | **67,3** | 100 | **74,0** | 99,2 | 100 | 100 | 100 | 99,2 | 100 | 99,4 | 98,2 |
| 15-007 | Canada | **67,3** | 100 | **74,0** | 99,2 | 100 | 100 | 100 | 99,2 | 100 | 99,4 | 98,2 |
| 2680 | Japan | **67,3** | 100 | **74,0** | 99,2 | 100 | 100 | 100 | 99,2 | 100 | 99,2 | 96,8 |
| 2725 | Japan | **67,3** | 100 | **74,0** | 99,2 | 100 | 100 | 100 | 99,2 | 100 | 99,2 | 96,8 |
| KG925 | Japan | **67,3** | 100 | **74,0** | 99,2 | 100 | 100 | 100 | 99,2 | 100 | 99,2 | 96,8 |
| 13-074 | USA | **67,3** | 100 | **74,0** | 99,2 | 100 | 100 | 100 | 99,2 | 100 | 99,4 | 98,2 |
| KG1616 | Japan | **67,3** | 100 | **74,0** | 99,2 | 100 | 100 | 100 | 99,2 | 100 | 99,4 | 98,2 |
