# Supplementary material for: Atypical Actinobacillus pleuropneumoniae serotype 12 strains with a higher virulence potential
Source: Vet Res. 2025 Jul 13;56:149. doi: 10.1186/s13567-025-01579-9 (PMC12255999; doi:10.1186/s13567-025-01579-9)
Supplement: Supplementary file 3 — Additional file 3. Actinobacillus pleuropneumonie LC-LPS ELISA serology results at euthanasia. Values are expressed as optical density values (414 nm). [file 13567_2025_1579_MOESM3_ESM.docx]

**Additional file 3**. ***Actinobacillus pleuropneumonie* LC-LPS ELISA serology results at euthanasia.** Values are expressed as optical density values (414_nm_.)

| **GROUP** | **Pig #** | **Serotypes**  **1 (9/11)** | **Serotype 2** | **Serotypes**  **3 (6, 8, 15, 17)** | **Serotype 5** | **Serotypes 7 (4,18)** | **Serotype 10** | **Serotype 12** | **Serotype 13** | **Serotype 14** |
| --- | --- | --- | --- | --- | --- | --- | --- | --- | --- | --- |
| CONTROL | 1 | 0,02 | 0,01 | 0,02 | 0,02 | 0,01 | 0,01 | 0,02 | 0,01 | 0,01 |
|  | 3 | 0,00 | 0,00 | 0,00 | 0,01 | 0,00 | 0,00 | 0,00 | 0,00 | 0,00 |
|  | 13 | 0,02 | 0,01 | 0,01 | 0,02 | 0,01 | 0,01 | 0,01 | 0,01 | 0,01 |
|  | 16 | 0,02 | 0,00 | 0,00 | 0,00 | 0,00 | 0,00 | 0,00 | 0,00 | 0,00 |
|  | 17 | 0,01 | 0,01 | 0,02 | 0,01 | 0,01 | 0,01 | 0,01 | 0,01 | 0,02 |
|  | 19 | 0,00 | 0,00 | 0,00 | 0,01 | 0,01 | 0,00 | 0,00 | 0,00 | 0,00 |
|  | 30 | 0,00 | 0,00 | 0,00 | 0,00 | 0,01 | 0,00 | 0,00 | 0,00 | 0,00 |
|  | 32 | 0,01 | 0,01 | 0,00 | 0,01 | 0,00 | 0,00 | 0,00 | 0,00 | 0,00 |
| 8329/85 STRAIN | 5 | 0,01 | 0,01 | 0,01 | 0,02 | 0,03 | 0,01 | **0,17** | 0,01 | 0,01 |
|  | 6 | 0,00 | 0,00 | 0,00 | 0,01 | 0,01 | 0,00 | **0,11** | 0,00 | 0,00 |
|  | 7 | 0,00 | 0,00 | 0,00 | 0,01 | 0,00 | 0,00 | 0,00 | 0,01 | 0,00 |
|  | 8 | 0,00 | 0,00 | 0,00 | 0,01 | 0,01 | 0,00 | 0,01 | 0,00 | 0,00 |
|  | 9 | 0,03 | 0,01 | 0,01 | 0,02 | 0,01 | 0,01 | 0,05 | 0,01 | 0,01 |
|  | 12 | 0,08 | 0,01 | 0,00 | 0,03 | 0,00 | 0,01 | **0,20** | 0,01 | 0,00 |
|  | 14 | 0,00 | 0,00 | 0,00 | 0,01 | 0,00 | 0,00 | 0,02 | 0,00 | 0,01 |
|  | 18 | 0,00 | 0,01 | 0,01 | 0,03 | 0,01 | 0,01 | **0,20** | 0,01 | 0,00 |
|  | 21 | 0,02 | 0,01 | 0,02 | 0,02 | 0,02 | 0,02 | **0,85** | 0,01 | 0,01 |
|  | 23 | 0,01 | 0,00 | 0,00 | 0,02 | 0,00 | 0,01 | 0,04 | 0,00 | 0,00 |
|  | 26 | 0,01 | 0,00 | 0,00 | 0,02 | 0,00 | 0,00 | 0,03 | 0,00 | 0,00 |
|  | 29 | 0,01 | 0,01 | 0,02 | 0,02 | 0,02 | 0,02 | 0,03 | 0,02 | 0,01 |
| 21-001-1 STRAIN | 2* | 0,01 | 0,00 | 0,00 | 0,01 | 0,00 | 0,00 | 0,00 | 0,00 | 0,00 |
|  | 4 | 0,01 | 0,00 | **0,20** | 0,01 | 0,00 | 0,00 | 0,00 | 0,00 | 0,01 |
|  | 10 | 0,00 | 0,01 | **0,41** | 0,01 | 0,00 | 0,06 | 0,00 | 0,01 | 0,00 |
|  | 11 | 0,00 | 0,00 | **0,41** | 0,02 | 0,00 | 0,00 | 0,08 | 0,00 | 0,00 |
|  | 15 | 0,01 | 0,00 | 0,00 | 0,00 | 0,00 | 0,00 | 0,00 | 0,00 | 0,00 |
|  | 20 | 0,00 | 0,00 | **0,28** | 0,00 | 0,01 | 0,03 | 0,01 | 0,02 | 0,00 |
|  | 22 | 0,00 | 0,00 | **0,25** | 0,01 | 0,00 | 0,00 | 0,01 | 0,00 | 0,00 |
|  | 24 | 0,01 | 0,00 | 0,09 | 0,00 | 0,00 | 0,00 | 0,04 | 0,00 | 0,01 |
|  | 25 | 0,02 | 0,02 | **0,46** | 0,02 | 0,02 | 0,02 | 0,02 | 0,02 | 0,02 |
|  | 27 | 0,01 | 0,00 | **0,51** | 0,01 | 0,01 | 0,01 | 0,01 | 0,01 | 0,00 |
|  | 28* | 0,01 | 0,00 | 0,01 | 0,01 | 0,00 | 0,00 | 0,02 | 0,00 | 0,00 |
|  | 31 | 0,00 | 0,00 | **0,21** | 0,01 | 0,00 | 0,00 | 0,00 | 0,00 | 0,00 |

*Animals euthanized 24 h post-infection
